# Supplementary material for: Food Insecurity Is Associated with Depression among a Vulnerable Workforce: Early Care and Education Workers
Source: Int J Environ Res Public Health. 2020 Dec 29;18(1):170. doi: 10.3390/ijerph18010170 (PMC7795637; doi:10.3390/ijerph18010170)
Supplement: Supplementary file 1 [file ijerph-18-00170-s001.pdf]

**Table S1.** Comparison of Seattle, WA and Austin, TX by Cost of Living, Demographic, and Child Care Center Employee Wages.

| Comparison Indicator [1] | Seattle, WA | Austin, TX |
|---|---|---|
| **Cost of Living** | | |
| Median Income per capita ($) | 39,152 | 34,959 |
| Median Income per household [2]($) | 75,331 | 67,195 |
| Overall Cost of Living [3] | 117 | 117 |
| Median Rent, 2014 ($) | 1084 | 1202 |
| **Demographic** | | |
| Population, *n* (% of state population) | 685,000 (10%) | 932,000 (4%) |
| % of Females | 50 | 50 |
| Median Age | 37 | 34 |
| % of Population with High School Graduate or Higher | 92 | 89 |
| Unemployment rate (%) | 4.0 | 2.9 |
| **Child Care Center Employee Wages** | | |
| Median Hourly Wage of Preschool/Childcare Administrators [4,5] ($) | 18.40 | 21.96 |
| Annual Mean Wage of Preschool/Childcare Administrators[4,5] ($) | 43,930 | 47,120 |
| Median Hourly Wage of Childcare Workers [4,6] ($) | 11.80 | 10.34 |
| Annual Mean Wage of Childcare Workers [4,6] ($) | 26,180 | 22,820 |

[1] Cells in grey reference 2015 ACS data for the cities' metropolitan statistical area (MSA): https://www.census.gov/programs-surveys/acs/.; Cells in blue reference 2010 Census data: https://www.census.gov/2010census/data/; Cells in green reference 2015 Bureau of Labor Statistics data (MSA): https://www.bls.gov/oes/current/oessrcma.htm; Cells in yellow reference 2014 ACS data from Civics Dashboard by OpenGov: http://www.civicdashboards.com/; Cells in pink reference http://www.bestplaces.net/compare-cities/costofliving; recommended by US State Dept at https://www.state.gov/m/fsi/tc/79700.htm; [2] This includes the income of the householder and all other individuals 15 years old and over in the household, whether they are related to the householder or not. Because many households consist of only one person, average household income is usually less than average family income. Although the household income statistics cover the past 12 months, the characteristics of individuals and the composition of households refer to the time of interview. The composition of most households was the same during the past 12 months as at the time of interview.; [3] Values reflect data updated 12/16. The total of all the cost of living categories weighted subjectively as follows: housing (30%), food and groceries (15%), transportation (10%), utilities (6%), health care (7%), and miscellaneous expenses such as clothing, services, and entertainment (32%). State and local taxes are not included in any category.; [4] by metropolitan region, May 2015; [5] Bureau of Labor Statistics (BLS) definition of preschool/childcare administrators: Plan, direct, or coordinate the academic and nonacademic activities of preschool and childcare centers or programs. Excludes "Preschool Teachers" (25-2011). [6] BLS definition of childcare workers: Attend to children at schools, businesses, private households, and childcare institutions. Perform a variety of tasks, such as dressing, feeding, bathing, and overseeing play. Excludes "Preschool Teachers, Except Special Education" (25-2011) and "Teacher Assistants" (25-9041).

**Table S2.** Additional characteristics of a cohort of 313 early care and education (ECE) providers in Washington and Texas participating in baseline data collection of a prospective study exploring the effects of wage on ECE provider health, by food security status, 2017.

| **Demographic Factor *** | **Food Secure (*n* = 185)** | **Low Food Security (*n* = 64)** | **Very Low Food Security (*n* = 64)** |
|---|---|---|---|
| **Marital Status, *n* (%)** | | | |
| Never Married | 78 (42%) | 28 (44%) | 40 (63%) |
| Now Married | 85 (46%) | 20 (31%) | 13 (20%) |
| Divorced, Widowed, Separated or Other | 22 (12%) | 16 (25%) | 11 (17%) |
| **Number of Children (<18) in Household, *n* (%)** | | | |
| 0 | 125 (68%) | 32 (50%) | 40 (63%) |
| 1 | 33 (18%) | 15 (23%) | 8 (13%) |
| 2 or more | 27 (15%) | 17 (27%) | 16 (25%) |
| **Individual Annual Income, $10,000, mean (SD)** | 31,783.5 (12,055) | 25,013.3 (10,254) | 23,543.2 (10,005) |
| **Household Income, mean (SD)** | 67,196.3 (45,483) | 41,856.7 (35,268) | 37,149.5 (24,695) |

* Percentages provided for each demographic factor reflect proportions of participants in each food security subgroup (i.e., for each demographic factor, percentages within the same column sum to 100%).

**Table S3.** Sensitivity analyses with logistic regression estimates (odds ratio) for the association between food insecurity and depression among a cohort of 313 early care and education (ECE) Providers in Washington and Texas *.

| | **Odds Ratio** | **95% Confidence Interval** | ***p*-Value** |
|---|---|---|---|
| **Sensitivity Analysis 3.2: Primary Model, replacing *number of children in household* with *Total number of People in Household (continuous)* (*n* = 299) ǂ** | | | |
| Low Food Security (*n* = 61) | Low Food Security (*n* = 61) | Low Food Security (*n* = 61) | Low Food Security (*n* = 61) |
| Very Low Food Security (*n* = 62) | Very Low Food Security (*n* = 62) | Very Low Food Security (*n* = 62) | Very Low Food Security (*n* = 62) |

* Presented values are estimated using logistic regression models for the association between food insecurity and depression. The primary model controls for age, marital status, birth country, race/ethnicity, number of children in the household, job title, average paid hours of work per week, highest level of education, total household income, and an indicator variable for study site. ǂ The reference group is participants who are food secure (*n* = 176).
